# Phytohormone Abscisic Acid Improves Spatial Memory and Synaptogenesis Involving NDR1/2 Kinase in Rats

**DOI:** 10.3389/fphar.2018.01141

**Published:** 2018-10-09

**Authors:** Juanjuan Liu, Xiaozhen Gu, Rongxin Zou, Wenping Nan, Shaohua Yang, Hui-Li Wang, Xiang-Tao Chen

**Affiliations:** ^1^School of Food Science and Engineering, Hefei University of Technology, Hefei, China; ^2^School of Pharmacy, Anhui Medical University, Hefei, China

**Keywords:** abscisic acid, spatial memory, synaptogenesis, the spine density, NDR1/2 kinase

## Abstract

The abscisic acid (ABA) is a phytohormone involved in plant growth, development and environmental stress response. Recent study showed ABA can also be detected in other organisms, including mammals. And it has been reported that ABA can improve learning and memory in rats. In this study, we attempted to investigate the effects of ABA on the alternation of dendritic spine morphology of pyramidal neurons in developmental rats, which may underlie the learning and memory function. Behavior tests showed that ABA significantly improved spatial memory performance. Meanwhile, Golgi-Cox staining assay showed that ABA significantly increased the spine density and the percentage of mushroom-like spines in pyramidal neurons of hippocampus, indicating that ABA increased dendritic spine formation and maturation, which may contribute to the improvement of spatial memory. Furthermore, ABA administration increased the protein expression of NDR1/2 kinase, as well as mRNA levels of NDR2 and its substrate Rabin8. In addition, NDR1/2 shRNA prohibited the ABA-induced increases in the expression of NDR1/2 and spine density. Together, our study indicated that ABA could improve learning and memory in rats and the effect are possibly through the regulation of synaptogenesis, which is mediated via NDR1/2 kinase pathway.

## Introduction

Abscisic acid ((2*Z*,4*E*)-5-[(1*S*)-1-hydroxy-2,6,6-trimethyl-4-oxocyclohex-2-en-1-yl]-3-methyl penta-2,4-dienoic acid, ABA) is an important phytohormone that regulates plant growth, development and environmental stress response (Li et al., 2000; Finkelstein, 2013). Under stress conditions, ABA accumulates in roots and leaves and triggers several adaptive responses (Arbona et al., 2017). Lately, ABA also proved to be secreted and active in mammals, where it stimulates the activity of innate immune cells, stem cells, insulin-releasing pancreatic β-cells, and serves as an endogenous hormone (Bruzzone et al., 2008, 2012; Scarfi et al., 2008; Magnone et al., 2009). Besides, it also holds promises as nutraceutical plant-derived compound which show potentially beneficial effects in human (De Flora et al., 2014). As a direct derivative of carotenoids, ABA and all-*trans* retinoic acid share a similar molecular structure, with a free carboxyl group at the end of the isoprene composed side-chain, which is the critical part of their bioactivity (Walker-Simmons et al., 1991; Moise et al., 2005). And retinoic acid (Vershinin, 1999; Nambara and Marion-Poll, 2005) as well as ABA (Qi et al., 2015; Naderi et al., 2017) has been reported to improve learning and memory and anxiety-like behavior.

Evidence suggests that spatial memory performance of rats is related to the level of granule cell neurogenesis (Drapeau et al., 2003). Dendritic spines, the bulbous protrusions that form the postsynaptic half of excitatory synapses, are one of the most prominent features of neurons (Alvarez and Sabatini, 2007). Composed of a round spine head and a thinner spine neck, dendritic spines serve as the point of contact between two neurons with an increased concentration of postsynaptic signaling components such as glutamate receptors (De Paola et al., 2006). Because they are easily observable features of neuronal morphology, dendritic spines have been stained and imaged in fixed brain tissue and changes in spine numbers and morphology have been associated with processes in development, as well as in learning and memory (Kasai et al., 2010; Bosch and Hayashi, 2012). For example, it was revealed that, in rat CA1 pyramidal cells, the volume of the spine is proportional to the postsynaptic density (PSD) area (Harris and Stevens, 1989) and the PSD area is itself proportional to the number of postsynaptic receptors (Nusser et al., 1998), therefore, the size of the spine head is likely to be directly proportional to the average reliability and strength of its synapse. In addition, spine stability is associated with long-term memory persistence (Yang et al., 2009). New dendritic spines are preferentially stabilized by a forelimb reaching training sessions (Xu et al., 2009).

Traditionally, dendritic spines are grouped into four classes: mushroom, stubby, thin, and filopodia (Yuste, 2011). The mushroom-like spine has a bulbous head attached to the dendrite by a narrow neck; thin spines have small heads and thin longnecks, whereas stubby spine with no neck, and filopodia with a longer neck and with no head enlargement. It is believed that long, thin spines are mostly immature spines, and they develop into the more mature mushroom-like spines (Smrt and Zhao, 2010).

In the current study, we tried to link the memory-improving effect of ABA to the alternation of dendritic spine morphology of pyramidal neurons in the hippocampus of developing rats.

A large number of factors, such as brain-derived neurotrophic factor BDNF (Chapleau et al., 2008; Neal et al., 2011), Shank, NDR1/2 kinase (Millward et al., 1998; Hergovich et al., 2006) regulate the growth of dendritic spines (Cline, 2001; Miller and Kaplan, 2003). Among these proteins, NDR1/2 kinase drew our attention because the evolutionarily conserved NDR1/2 kinase pathway, important for polarized growth from yeast to mammals, controls dendrite growth and morphology (Hergovich et al., 2006; Emoto, 2011). Rabin8, a GDP/GTP exchange factor (GEF) of Rab8 GTPase, is one of the substrates of NDR1/2 kinase, which participates in regulating dendrite growth, especially spine growth (Ultanir et al., 2012). It has been shown that NDR1/2 kinase can be activated by calmodulin and s100 (an intracellular second messenger Ca^2+^, being capable of modulating NDR activity) (Peng et al., 2002). And NDR1/2 kinase substrate AAK1 contributes to dendrite growth regulation, and Rabin8 regulates spine development (Ultanir et al., 2012).

In this study, we attempted to investigate the effects of ABA on dendritic spine formation and maturation in related to function of learning and memory. In addition, we explored NDR1/2 kinase protein expression, as well as the NDR1, NDR2, and Rabin8 transcription mRNA level, which may contribute to the morphological change of dendritic spines.

## Materials and Methods

### Animals and Experimental Design

Sprague-Dawley rats were obtained from the Laboratory Animal Centre, Anhui Medical University, China. The study was executed in accordance with the Guide for the Care and Use of Laboratory Animals, 8th edition (National Institutes of Health 2011) and was approved by the Institutional Animal Care and Use Committee of Anhui Medical University, China. Rats were individually housed in a temperature (20 ± 3°C) and humidity (50 ± 10%) controlled environment on a 12–12 h light-dark cycle with free access to food and water.

The pups of rat were divided into the control group, Low dose group (ABA 5 mg / kg / day), Medium dose group (ABA 25 mg / kg / day), and High dose group (ABA 50 mg / kg / day). There were 9 animals in each group, 5 in male and 4 in female.

Abscisic acid (Sigma, St. Louis, MO, United States) was dissolved in the vehicle of sterile saline solution (0.9% w/v sodium chloride) with dimethyl sulphoxide (DMSO) at a ratio of 1:1 (v/v). Because the postnatal day (PND) 7–21 is believed to be the sensitive periods in the development of the brain and behavior (Spear, 2000; Cai et al., 2010), the rats were given daily intraperitoneal injections of ABA form PND 7 to the end of the study, while the rats in control group received the vehicle. They were then subjected to Morris water maze (MWM) tests from PND56 to PND62. 3 days after the last MWM test, the rats were killed under deep anesthesia with CO_2_ and brains were cut into two hemispheres longitudinally; the right part was prepared for morphological staining, and the left one for special proteins expression measuring and real time fluorescence quantitative PCR assay.

### MWM Tests

The MWM test was used to evaluate long-term spatial learning and memory in rodents (Taghizadeh et al., 2011). The experimental device consisted of a circular tank with a diameter of 160 cm and depth of 70 cm, containing water hold constant at 23 ± 1°C. A hidden platform was placed on a fixed location in the center of one of four supposed quadrants of the pool. During the acquisition (learning) phase rats were trained to swim to the hidden platform with its top surface submerged 1.5 cm below the water level. For each trial, the animals were released from a different position in the water maze. Each rat was given 90 s to find the hidden platform and was allowed to stay on the platform for 30 s. Each rat performed four trials daily for 5 days. The latency to find the platform in the acquisition phase was recorded each day to assess learning performance. During the probe test session (on the 6th day), the rats were given a 90 s retention trial in which the platform was removed. The time spent in the target quadrant, the latency and frequency to choose the target quadrant, and the average distance to the former platform were measured to assess the characterization of memory.

### Western Blotting Assay

Proteins were extracted as described previously (Liu et al., 2016). Briefly, hippocampus was homogenized and dissolved in ice-cold lysis buffer (PBS, pH 7.4) containing a cocktail of protein phosphatase and protease inhibitors (21 μg/ml aprotinin, 0.5 μg/ml leupetin, 4.9 mM MgCl2, 1mM sodium-Meta-vanandante, 1% Triton X-100, and 1mM PMSF) to avoid de-phosphorylation and degradation of proteins. Subsequently, all the samples were centrifuged at 14000 ×*g* at 4°C for 7 min followed by collecting supernatant which was assayed for total protein concentration. Proteins were separated in 8.5% SDS–PAGE gel, and then transferred to PVDF membrane, blocked with 5% non-fat dry milk, followed by incubation with primary antibodies overnight at 4°C. Then membranes were washed for three times, incubated with secondary antibody and then processed for visualization using the enhanced chemiluminescence immuno-blotting detection system. All results were normalized against GAPDH. GAPDH was purchased from Abcam, ab9484, monoclonal, 1:5000, NDR1/2 was from Santa cruz, sc271703, monoclonal, 1:2000.

### Golgi-Cox Staining Assay

The brain was processed by Golgi-Cox staining method (Liu et al., 2015). In brief, brains were stored in a dark place for 2 days (37°C) in Golgi-Cox solution, and then sectioned at 200 μm in 6% sucrose with a vibratome (VT1200, Leica, Germany). All hippocampal sections were collected on 2% gelatin-coated slides. Then, the slices were stained with ammonia for 60 min, washed with water for three times, followed by Kodak Film Fix for 30 min, and then washed with water, dehydrated, cleared, and mounted using a resinous medium. The neurons in hippocampus were imaged with a Nikon widefield microscope (Eclipse 80i) by using a 40 × objective. Then, Neurons that had a pyramidal-shaped cell body and a clear secondary dendrite were chosen for analysis.

The total dendritic protrusion density was counted according to a previously reported method (Orlowski and Bjarkam, 2012) and the percentage of mushroom like spines on pyramidal was measured.

Spines were counted in the Image J. The spines counted in the present study were on 2 – 3 stretches of the secondary dendrite about 10 μm in length. About 6 – 8 neurons from one animal were selected to analyze the spine morphology and dendrite number. The spine density was estimated as the number of spines on each terminal dendrite per 10 μm.

The neck length was determined as the distance between the branch point from the parent dendrite to the starting point of the spine head (or to the end-point of the protrusion in case of filopodium-like long spines). According to their morphology, protrusions were distinguished. (1) mushroom spines with a short neck (<1 μm) and a head; (2) stubby spines with a head but without a neck; (3) long spines with a long neck (>1 μm) and small heads; and (4) filopodia with no detectable head. Counting the number of protrusions that belong to the respective categories.

### Primary Neuronal Cultures and NDR shRNA Transfection

Primary hippocampal cultures were prepared from the brains of Rats at PND 0 (Wang et al., 2011). Briefly, hippocampi were dissociated by enzymatic digestion in 0.03% trypsin for 19 min at 37°C and then triturated with a fire-polished Pasteur pipette. Neurons were plated on poly-L-lysine (0.5 mg/ml; Sigma-Aldrich)-treated 25 mm glass coverslips at a density of 100,000 cells per coverslip.

The cultured hippocampal neurons were transfected with shRNA (Biomics Biotechnologies, Guangzhou, China) using Lipofectamine 2000 (Invitrogen) at DIV 9. The Neurons was administered 10 μM ABA for 48 h at DIV14.

### Real-Time Fluorescence Quantitative PCR

The total RNAs in developmental hippocampus were extracted using the RNA kit (Axygen, Silicon Valley, CA, United States) from the hippocampus. Subsequently, the primer OligodT were used to complete the reverse transcription reaction according to the manufacturer’s instructions (TransGene, Shanghai, China), resulting in the first strand of total cDNA. The 20 μL reaction pool of RTFQ PCR was composed of 10 μL of SYBR premix Extaq; 0.8 μL of forward and reverse primer each; 2 μL of cDNA template (10 times dilution); and 6.4 μL of deionized water. The primers used in this protocol were listed as follows:

GGGTTAAGGGTGATTGATGTTCG-AGGCACCTCTATCTCCTTCGCA for NDR1;
GAACGGAGCCTGGGTAGTGA-AAAGGTTGTCTGGCTTGATGTC for NDR2;
GTTCCAGAGCCAGCATCATCG-TCATCGTTGCCAGCAGAAGC for Rabin8;
CTGTGCTATGTTGCCCTAGACTTC-CATTGCCGATAGTGATGACCTG for r-Actin.

The real-time fluorescence PCR system was from Roche (Roche Lightcycler 96). The reaction procedure was set as one cycle of 95°C for 10 s, 40 cycles of 95°C for 10 s, and 60°C for 30 s, followed by the melting stage of 95°C for 10 s, 65°C for 60 s, and 97°C for 1 s, then the cooling stage of 37°C for 30 s. The transcription levels were calculated as the amounts relative to that of r-Actin under the same conditions.

### Statistical Analysis

All data were expressed as mean ± SEM. One-way ANOVA was applied to the data of dendritic spine density, Western blot protein assay, quantitative PCR assay and the data of probe trial in MWM tests. Two-way ANOVA was applied to the data during acquisition training in MWM tests. Difference between groups was then tested using Fisher’s protected least significant difference (PLSD) with 95% confidence. A value of *p* < 0.05 was considered to be statistically significant.

## Results

### ABA Improved Spatial Memory in SD Rats

**FIGURE 1:**
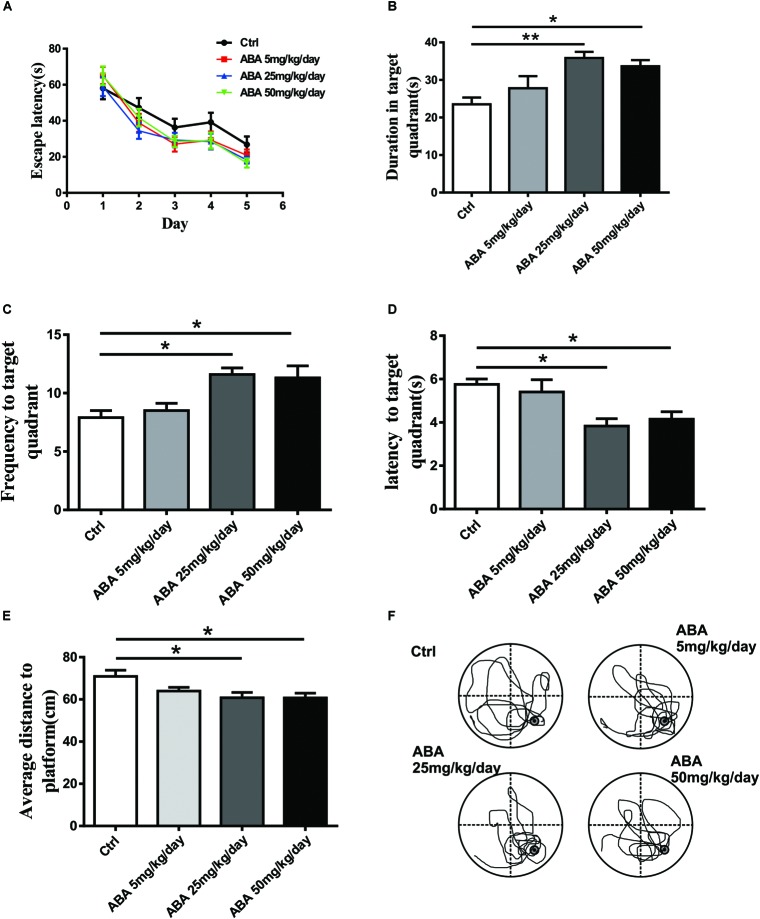
Effect of ABA on MWM performance in rats. **(A)** The escape latency to locate the platform over the 5 days of acquisition, **(B)** The time spent in the target quadrant, **(C)** The frequency entering the target quadrant, **(D)** The latency entering the target quadrant, **(E)** The average distance to the former platform, and **(F)** Representative swim paths of control and ABA rats in the probe test. Data are shown as means ± SEM (*n* = 9 rats per group, ^∗^*P* < 0.05, and ^∗∗^*P* < 0.01).

Morris water maze test was employed to assay the effect of ABA on spatial learning and memory in SD rats. The Two-way ANOVA (4 different treatments × 5 training days, with repeated measures on days) revealed that spatial learning improved over time in all groups [*F*(4,160) = 40.82, *P* < 0.0001]. Non-significant differences were found between the ABA and control rats in acquiring spatial information, whereas, ABA rats showed a trend toward shorter escape latency before locating the platform [*F*(3,160) = 2.452, *P* = 0.0645]. (**Figure 1A**). Meanwhile, probe tests showed that ABA significantly increased the time spent in the target quadrant [*F*(3,32) = 5.851, *P* = 0.0026] and the number of crossing platform [*F*(3,32) = 5.725, *P* = 0.0030] and decreased the latency to locating the target quadrant [*F*(3,32) = 5.809, *P* = 0.0027] and the average distance to the former platform [*F*(3,32) = 7.794, *P* = 0.0005] (**Figures 1B–F**). It suggested that administration of ABA could improve spatial memory performance in SD rats.

### ABA Changed the Spine Density and the Percentage of Mushroom-Like Spines

**FIGURE 2:**
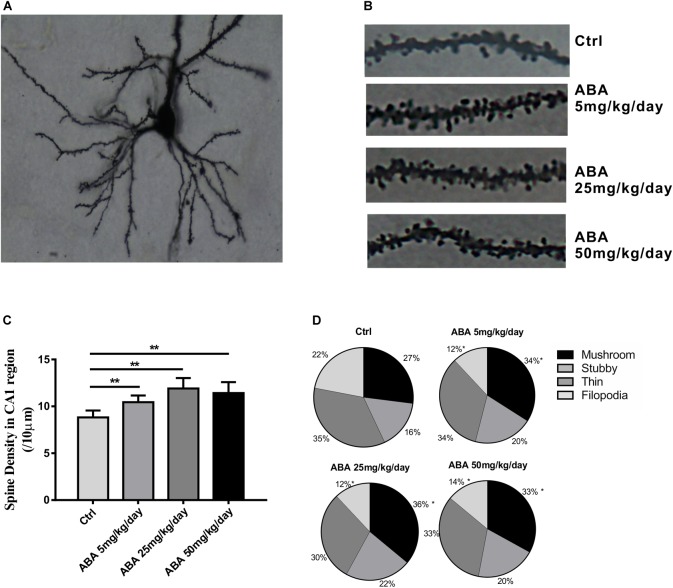
Effects of ABA on spine density and the percentage of mushroom-like spines. **(A)** Representative images of Golgi-Cox impregnated CA1 pyramidal neuron, **(B)** Representative dendritic shaft with spines of hippocampal neurons, **(C)** The changes of dendritic spine density (/10 μm), and **(D)** The percentage of different types of spines in CA1 pyramidal neuron of rats. Data are shown as means ± SEM (*n* = 40∼50 neurons per group in 9 rats, ^∗^*p* < 0.05 vs Ctrl, and ^∗∗^*p* < 0.01 vs Ctrl).

To explore the effect of ABA on spine formation, the spine density in the hippocampal CA1 areas was measured. Compared with the control group, Low, Medium, and High doses of ABA administration increased the dendritic spine density in hippocampal CA1 pyramidal neurons [*F*(3,196) = 44.72, *P* < 0.0001] (**Figures 2A–C**). It suggested that ABA increased dendritic spine formation. Mushroom-like dendritic spines are relatively stable and mature in four types (Yuste, 2011). The percentage of mushroom-like spines was measured to explore the effects of ABA on spine maturation. Compared with control group, the percentage of mushroom shaped spines in CA1 neurons increased significantly in ABA groups [*F*(3,196) = 14.71, *P* < 0.0001] while the percentage of filopodia type spines decreased significantly [*F*(3,196) = 101.9, *P* < 0.0001] (**Figure 2D**). It suggested that ABA possibly increased the stable dendritic spines due to a transformation of less mature spines.

### ABA Increased the Protein Expression of NDR1/2 Kinase in Hippocampus

**FIGURE 3:**
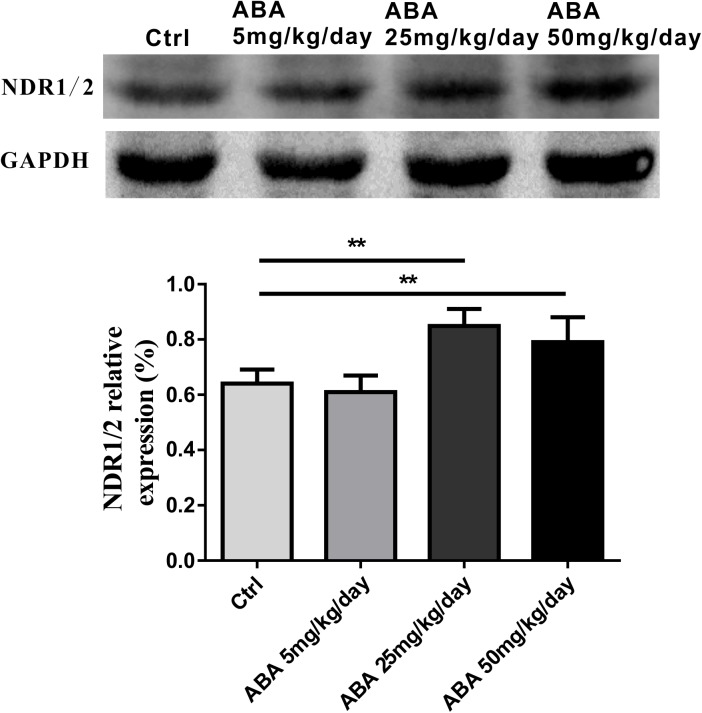
Effects of ABA on NDR1/2 protein expression in hippocampus. Representative immunoblot bands and histograms plot showing NDR1/2 protein expression in hippocampus. GAPDH was used as a loading control. The optical density of bands was quantified by Image-J software. Data are shown as means ± SEM (*n* = 9 rats per group and ^∗∗^*p* < 0.01).

NDR1/2 kinase plays an important role in the development of neuronal dendrites and dendritic spines, making it a potential candidate for studying the changes of dendritic spines after ABA administration. It is seen that after administrating with ABA, NDR1/2 kinase expression was significantly increased [*F*(3,32) = 26.31, *P* < 0.0001] (**Figure 3**). Our study provided the preliminary indication that NDR1/2 kinase could be involved in the process of ABA on the growth of neuronal dendrites.

### ABA Up-Regulated mRNA Levels of NDR2 and Its Substrate Rabin8 in Hippocampus

**FIGURE 4:**
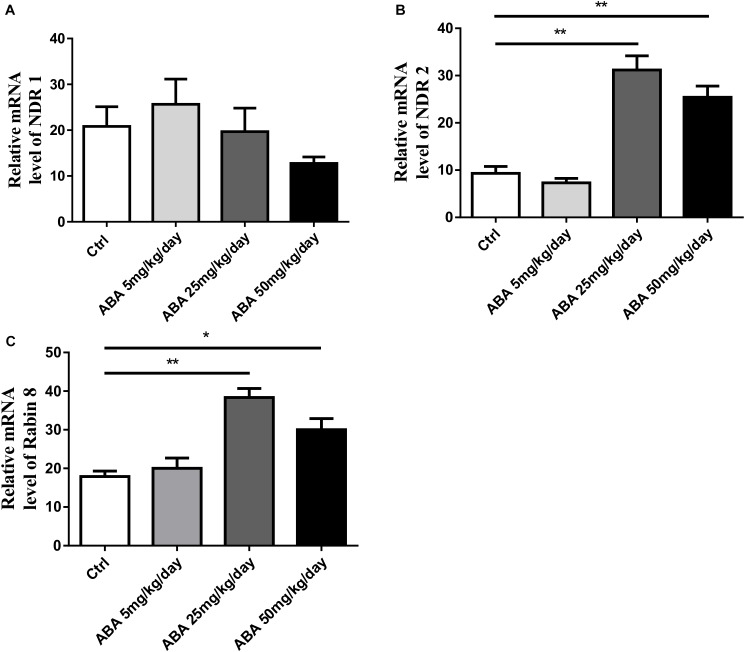
Effects of ABA on transcription level of NDR1, NDR2 and Rabin8 in hippocampus. RT-PCR exhibits the transcription level. **(A)** NDR1, **(B)** NDR2, and **(C)** Rabin8. The transcript amount was standardized by the amount of r-Actin in each sample. All the results were calculated as averages of triplicate experiments. Data are expressed as mean ± SEM (*n* = 9 per group, ^∗^*P* < 0.05, and ^∗∗^*P* < 0.01).

To further explore the role of NDR1/2 kinase pathway in regulating dendrite growth by ABA, we then investigated the alternation of NDR1, NDR2, and Rabin8 mRNA levels by real-time fluorescence PCR. The results showed that ABA significantly increased the expression of NDR2 [*F*(3,8) = 31.04, *P* < 0.0001] (**Figure 4B**) accompanied with increased Rabin8 mRNA levels in the hippocampus [*F*(3,8) = 15.62, *P* = 0.0010] (**Figure 4C**), while ABA failed to affect NDR1 expression [*F*(3,8) = 1.005, *P* = 0.4390] (**Figure 4A**). These results suggested the role of NDR1/2 kinase pathway in the process of regulating dendritic length and mushroom spine formation regulated by ABA.

### NDR1/2 shRNA Prohibited ABA-Induced Increase in Spine Density and the Expression of NDR1/2

**FIGURE 5:**
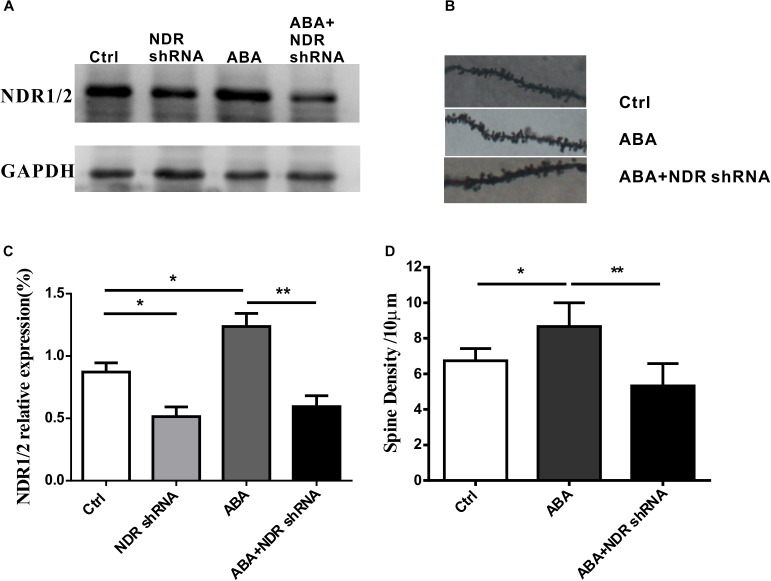
NDR1/2 shRNA prohibited ABA-induced increase in the expression of NDR1/2 and spine density. Hippocampal neurons were transfected with NDR1/2 shRNA to detect the role of ABA in dendritic spine formation by the NDR1/2 kinase pathway. **(A,C)** The expression of NDR1/2 kinase in cultured neuron with NDR1/2 shRNA, **(B)** representative dendritic shaft with spines of hippocampal neurons, and **(D)** the spine density in cultured neuron with NDR1/2 shRNA. All experiments were repeated three times from independent cultures. (*n* = 40∼45 neurons per group, ^∗^*p* < 0.05, and ^∗∗^*p* < 0.01).

To further validate the role of ABA in dendritic spine formation by the NDR1/2 kinase pathway, NDR1/2 shRNA plasmids was transfected into cultured hippocampal neurons. As shown in **Figure 5C**, NDR1/2 shRNA significantly inhibited the expression of NDR1/2 (Ctrl vs. NDR1/2 shRNA, *P* < 0.05). ABA (10 μM, 48 h) treatment significantly raised the NDR1/2 expression (Ctrl vs. ABA, *P* < 0.05), however, the increase was prohibited by lipo-transfection of NAD1/2 shRNA to ABA treated cells (ABA vs. ABA + NDR shRNA, *P* < 0.01) (**Figures 5A,C**). Also, for the spine density, ABA increased the spine density, whereas this increase was prohibited with NAD1/2 shRNA (**Figures 5B,D**). These data approved that the NDR1/2 kinase pathway could be possibly involved in the regulatory role of ABA for the development of dendritic spines in the brain neurons.

## Discussion

Abscisic acid is a classical plant hormone. The presence of ABA in the brain tissues of rats and pigs has been reported in 1986 (Le Page-Degivry et al., 1986). It has been indicated that exogenous administration of ABA improves learning capability and mood state of rats (Qi et al., 2014, 2015; Naderi et al., 2017). So, it may be a potential therapeutic molecule of memory enhancer, which could be used in memory deficits disease, e.g., Alzheimer disease, in future study. Thus, we intended to explore the effect of ABA on brain, especially on the function of learning and memory. To address the issues, we conducted MWM trials in SD rats, and our results showed that long-term management of ABA can improve the ability of memory. Further, we found that ABA promoted the dendritic spine formation, as well as the maturation process of dendritic spine. In addition, accompanied with the morphological changes of spine, we also found the increase of NDR1/2 kinase expression, as well as its substrate Rabin8, in the hippocampus, which indicates that the NDR1/2 kinase pathway may be involved in the regulation of ABA on synaptogenesis of dendritic spine in brain neurons.

We used adolescent rats in our study because adolescence and puberty are the major and prominent periods of pronounced developmental changes in the brain, particularly in relation to the motivational and cognitive behaviors (Spear, 2000; Cai et al., 2010). Therefore, this age group may be particularly vulnerable to the effects of drugs and could serve as an ideal group for this experiment. The MWM test is a widely accepted model for evaluating long-term spatial learning and memory (Sodhi and Singh, 2013). It requires the use of spatial environmental cues to form a cognitive map that the animal can use to locate the platform (Vorhees and Williams, 2006). During the training trials of the MWM test, ABA rats failed to display significant improvements in learning compared with the control rats. However, in the probe test of MWM, ABA administration significantly increased the duration spent in the target quadrant and the frequency of locating the target quadrant. Further, it decreased the latency to find the target quadrant and average distance to the former platform. These results indicated that administration of ABA can benefit memory performance, which is consistent with previous studies (Qi et al., 2015; Naderi et al., 2017), although, the underlying mechanisms for memory improvement of ABA requiring further in-depth studies.

The synapses, composed of a varicosity or bouton from a presynaptic neuron that communicates with a dendritic spine of the postsynaptic neuron, comprise the neural network that is essential for complex behavioral phenomena and cognition (Warburton and Brown, 2015). A dendritic spine is a small membranous protrusion from a neuron’s dendrite that typically receives input from a single axon at the synapse. Dendritic spines are very “plastic,” that is, spines change significantly in shape, volume, and number in small time courses (Alvarez and Sabatini, 2007). Spine plasticity is implicated in motivation, learning, and memory. In particular, spine alternation may play an important role in the maintenance of memory (Xu et al., 2009). And dynamic changes in the number and structure of dendritic spines are a hallmark of synaptogenesis (Maiti et al., 2015). In this study, we investigated the morphology of dendritic spines in the hippocampus of the brain, which would help us to understand how ABA affects hippocampal synaptogenesis and memory function. Golgi-cox staining showed that ABA administration increased dendritic spine density and the percentage of mushroom dendritic spine, it implies that ABA could improve spine formation and maturity to refine synaptic efficacy, thus improve learning and memory function.

It is well known that protein kinases play an important role in the regulation of life processes (Cline, 2001; Miller and Kaplan, 2003). In animals, the NDR1/2 kinase pathway is significantly involved in the development of neurons and the maturation of dendrites and dendritic spines (Ultanir et al., 2012). Therefore, we were keen to detect the protein expression of NDR1/2 kinase in the hippocampus of rats, and the results showed that ABA could increase the expression of NDR1/2 kinase at the protein level in the brain of rats. To further investigate the regulation of NDR1/2 kinase pathway in the process of ABA, the mRNA levels of NDR1, NDR2 and their substrate Rabin8 were detected. It was observed that ABA could mainly affect the expression of NDR2 mRNA levels with significant increase. The role of mammalian NDR1 and 2 in development has not yet been fully established. NDR1 has been shown being not crucial for mammalian development, because NDR1-deficient mice are viable and fertile and loss of NDR1 is compensated for by the elevation of total NDR2 protein levels, as observed in Ndr1^-/-^ mouse embryonic fibroblasts (Hergovich et al., 2006). Our result gives a preliminary clue to the role of NDR2 in neuronal morphogenesis affected by ABA. In addition, the expression level of Rabin8 mRNA in the downstream substrate of NDR1/2 kinase was also increased under ABA treatment. These results demonstrated that ABA could regulate the NDR1/2 kinase pathway from transcriptional levels.

To further test the role of ABA in dendritic spine formation by the NDR1/2 kinase pathway, we transfected NDR1/2 shRNA plasmids into cultured hippocampal neurons. The expression of NDR1/2 kinase was observed to be block by NDR1/2 shRNA in both ABA-treated and non-treated neurons. Meanwhile, the increased spine density in ABA treated neuron was also inhibited with DNR1/1 shRNA. These data verify the indication that the NDR1/2 kinase pathway could possibly participate in the regulation of dendritic spine development by ABA.

To summarize, ABA could improve learning and memory performance in rats. The results are possibly through the regulation of synaptogenesis, which is mediated via NDR1/2 kinase pathway. We postulate that ABA could have a potential therapeutic value in improving cognitive impairment.

## Author Contributions

X-TC and H-LW conceived and designed the experiments. JL, XG, WN, and SY performed the experiments. JL and RZ analyzed the data and wrote the paper. XG and X-TC revised the paper.

## Conflict of Interest Statement

The authors declare that the research was conducted in the absence of any commercial or financial relationships that could be construed as a potential conflict of interest.
